# MinION Nanopore-based detection of *Clavibacter nebraskensis*, the corn Goss’s wilt pathogen, and bacteriomic profiling of necrotic lesions of naturally-infected leaf samples

**DOI:** 10.1371/journal.pone.0245333

**Published:** 2021-01-22

**Authors:** Renlin Xu, Lorne Adam, Julie Chapados, Atta Soliman, Fouad Daayf, James T. Tambong

**Affiliations:** 1 Ottawa Research and Development Centre, Agriculture and Agri-Food Canada, Ottawa, Ontario, Canada; 2 Department of Plant Science, University of Manitoba, Winnipeg, Manitoba, Canada; Universidade de Coimbra, PORTUGAL

## Abstract

The Goss’s bacterial wilt pathogen, *Clavibacter nebraskensis*, of corn is a candidate A1 quarantine organism; and its recent re-emergence and spread in the USA and Canada is a potential biothreat to the crop. We developed and tested an amplicon-based Nanopore detection system for *C*. *nebraskensis* (Cn), targeting a purine permease gene. The sensitivity (1 pg) of this system in mock bacterial communities (MBCs) spiked with serially diluted DNA of *C*. *nebraskensis* NCPPB 2581^T^ is comparable to that of real-time PCR. Average Nanopore reads increased exponentially from 125 (1pg) to about 6000 reads (1000 pg) after a 3-hr run-time, with 99.0% of the reads accurately assigned to *C*. *nebraskensis*. Three run-times were used to process control MBCs, Cn-spiked MBCs, diseased and healthy leaf samples. The mean Nanopore reads doubled as the run-time is increased from 3 to 6 hrs while from 6 to 12 hrs, a 20% increment was recorded in all treatments. Cn-spiked MBCs and diseased corn leaf samples averaged read counts of 5,100, 11,000 and 14,000 for the respective run-times, with 99.8% of the reads taxonomically identified as *C*. *nebraskensis*. The control MBCs and healthy leaf samples had 47 and 14 Nanopore reads, respectively. 16S rRNA bacteriomic profiles showed that *Sphingomonas* (22.7%) and *Clavibacter* (21.2%) were dominant in diseased samples while *Pseudomonas* had only 3.5% relative abundance. In non-symptomatic leaf samples, however, *Pseudomonas* (20.0%) was dominant with *Clavibacter* at 0.08% relative abundance. This discrepancy in *Pseudomonas* abundance in the samples was corroborated by qPCR using EvaGreen chemistry. Our work outlines a new useful tool for diagnosis of the Goss’s bacterial wilt disease; and provides the first insight on *Pseudomonas* community dynamics in necrotic leaf lesions.

## Introduction

The emergence or re-emergence and rapid spread of bacterial plant diseases are a global biothreat to crop biosecurity. Increased prevalence and rapid spread of phytobacterial diseases are facilitated by environmental changes, increased international trade and immigration as well as the emergence of new virulence traits [1, 2]. The Gram-positive bacterial genus *Clavibacter* (family Microbacteriaceae) comprises of six core species [3], all of which are important phytopathogens on specific agricultural crops [4, 5]. Four of these species are classified as quarantine organisms due to the high economic threat they pose [6, 7]. *Clavibacter nebraskensis* [3] (formerly *C*. *michiganensis* subsp. *nebraskensis*) is the causal agent of the Goss’s bacterial wilt and blight disease of corn/maize (*Zea mays* L.). The first report of the disease was in south-central Nebraska (U.S.A) and neighboring states in 1969 [8] but in recent years it has re-emerged and spread within the USA as well as Canada [5, 9, 10]. In 2015, Singh et al. [11], reported the first account of the Goss’s wilt disease caused by *C*. *nebraskensis* in Louisiana. As this pathogen has the potential to invade seeds with no characteristic symptoms on seeds, as well as the ability to cause latent infections [12], Paul and Smith [13] proposed *C*. *nebraskensis* as a candidate A1 quarantine organism. This could have potential regulatory challenges for export countries. Rapid detection and accurate identification of the causal agents are key to developing reliable management strategies to mitigate crop yield losses and provide acceptable regulatory export/import assurance of minimal risk for spread of the disease to regions with no recent reports.

Compared to morphological and serological methods, DNA-based techniques provide a rapid and more reliable detection and identification of *C*. *nebraskensis* (Cn) on corn. Several conventional PCR assays have been reported. An assay based on specific random fragment length polymorphism patterns was reported by Waleron et al. [14]. Also, McNally et al. [15] reported a PCR-mediated detection assay using primers derived from a predicted gene; Feng et al. [16] developed a nested-PCR based on 16S-23S intergenic transcribed spacers (ITS); while Baek et al. [17] developed specific primers that yielded 500–564 bp fragments. These techniques require post-PCR handling to visualize the results and as such are prone to errors [5]. Also, a couple of real-time PCR-based methods have been developed for the detection of Cn [5, 18, 19]. Even though, real-time PCR-based systems provide a better alternative to conventional PCR assays, there are some inherent limitations e.g. the number of fluorescent dyes which can limit the number of target pathogens identified simultaneously.

Rapid advances in new next-generation DNA sequencing platforms is facilitating the identification of microbial species [2, 5, 20]. One of the most predominant systems is the MinION Nanopore sequencing platform (Oxford Nanopore Technologies (ONT)). Nanopore sequencing enables direct sequencing of native DNA by measuring voltage changes when a single DNA molecule passes through a protein Nanopore embedded in a membrane on a flowcell [21]. The decoding of the resulting signal determines the specific nucleic acid DNA sequence [21]; https://nanoporetech.com/applications/dna-nanopore-sequencing). The relatively low cost, portability and real-time data analysis of the Nanopore sequencing platform are attractive advantages while low read accuracy (90%) is its current main drawback [22]. Given its transportability, the MinION platform is becoming a useful tool for on-site sample sequencing for rapid microbial identification in diverse environments, e.g. microbial paleo mats in the Antarctic [23]; bacterial identification in clinical samples within 6 h [24], or field detection of cassava mosaic virus in Africa in less than 4 h [25]. Of all the studies that used the Nanopore technology for microbial identification, the majority have been in medical clinical samples [26, 27]. The use of this new technology to detect plant pathogens is still very limited. In addition to the virus testing by Boykin et al. [25], Chalupowicz et al. [28] demonstrated the detection of several phytobacterial pathogens using Nanopore technology while Hu et al. [2] applied the technique to the diagnosis of fungal wheat diseases caused by *Zymoseptoria tritici*, *Puccinia striiformis* f. sp. *tritici* and *Pyrenophora tritici repentis* in a nursery.

This article describes a Nanopore-based detection system for the Goss’s bacterial wilt and leaf blight pathogen, *C*. *nebraskensis*, by targeting a purine permease gene. The assay allowed for the detection of the pathogen in (1) a mock bacterial community (MBC) that included all known corn bacterial pathogens as well as other *Clavibacter* species to test its reliability; (2) serially diluted Cn DNA to determine the sensitivity/detection limit; and (3) Cn naturally-infected corn leaves versus healthy leaves. In addition, Nanopore runs of 3, 6, and 12 h were evaluated to determine the best run time. Also, bacteriomic analysis targeting the 16S rRNA gene was conducted to determine bacterial community profiles in diseased versus healthy leaf samples. Nanopore-based bacteriomic analysis revealed a significantly low *Pseudomonas* community in infected corn leaves that showed a high *Clavibacter* community as indicated by Nanopore reads. The decrease in *Pseudomonas* community population was confirmed by qPCR using *Pseudomonas*-specific primers targeting the 16S rRNA genes [29].

## Materials and methods

### Bacterial strains, plant material and DNA extraction

Twenty-eight bacterial strains, used as mock community, consist of other *Clavibacter* species/subspecies; seven corn bacterial pathogens (*Pseudomonas syringae* pv. *syringae*, *Xanthomonas vasicola* pv. *holcicola*, *Pantoea stewartii*; *Enterobacter cloacae* pv. *dissolvens*, *Pectobacterium carotovorum* pv. *carotovorum*, *Dickeya zeae* and *Panotea ananatis*); known endophytes/saprophytes, mainly of the *Pseudomonas fluorescens*/*Pseudomonas putida* subgroups, *Pantoea vagans* and *Pantoea agglomerans*. In addition, four *Streptomyces spp*. (*S*. *scabies*, *S*. *bottropensis*, *S*. *luridiscabiei* and *S*. *tubercidicus*) were used to represent distant bacterial species. With the exception of *Pectobacteriun spp*. and *Streptomyces spp*., all strains were cultured in Luria-Bertani (tryptone at 10 g/L, yeast extract at 5 g/L and NaCl at 10 g/L) or nutrient broth media as previously described [5]. Strains of *Pectobacteriun spp*. and *Streptomyces spp*. were cultured as previously described [30]. Bacterial stock cultures were maintained on the same medium amended with 25% glycerol (v/v). Genomic DNA was extracted from each strain using the Wizard SV Genomic DNA purification system (Promega Corp., Canada). DNA concentration was determined using the Qubit 4 fluorometer (ThermoFisher Scientific, Canada) and purified DNA was stored at -20°C.

In the 2015 and 2016 corn growing seasons, 25 diseased and healthy corn leaves were collected in Manitoba and shipped to the Ottawa Bacteriology lab and processed as indicated previously [5]. Total DNA was extracted from punched leaf discs (4 discs/leaf sample) for 16 leaf samples consisting of 8 Cn-symptomatic and 8 healthy samples previously indicated to be Cn-positive and Cn-negative using a multiplex TaqMan real-time PCR [5]. Total DNA was extracted from these 16 samples with Ultraclean Soil DNA isolation kits (Mo Bio Laboratories) as indicated by the manufacturer. DNA quality and concentration were determined using agarose (1%) electrophoresis and Qubit 4 fluorometer (ThermoFisher). Also, the total DNA was evaluated for potential PCR inhibition using the IC-65for/IC-65rev primer pair with the hex-labelled probe (IC-88pb) as previously described [30]. The total DNA of 4 leaf samples were pooled to constitute two diseased and two healthy samples processed by PCR and Nanopore sequencing.

### Gene selection and primer design for nanopore detection of Cn

The genomes of five core *Clavibacter* species (*C*. *michiganensis*, *C*. *sepedonicus*, *C*. *insidiosus*, *C*. *tessellarius* and *C*. *nebraskensis*) were annotated on RAST [31] and comparative genomics analysis (sequence comparison tool) was performed as described previously [4] to identify unique genomic regions for primer design. A purine permease gene fragment was identified as a candidate for Nanopore-based detection of Cn. The gene is located within the 258009–258981 region of the genome (HE614873) of *C*. *nebraskensis* NCPPB 2581^T^. The locus tag for this fragment is CMN_00274. Primers were designed and tested for specificity, as reported previously [5, 32]. The primers were checked for melting temperature (Tm), dimer or hairpin formation using an oligonucleotide properties calculator [33]. The Nanopore-specific nucleotide sequences were fused to the 5’-end of the designed purine permease primers as recommended by Oxford Nanopore Technologies. Primers were synthesized by Integrated DNA Technologies (Canada).

### PCR amplification and Nanopore library construction and sequencing

Primer sets 16S27F/16SR1495 (5’-AGAGTTTGATCMTGGCTCAG-3’ / 5’- TACGGYTACCTTGTTACGACTT-3’) [34] and Cn_pPer11F/Cn_pPer986R (5’-CCTCGACCACCGCCGCGA-3’ / 5’-CGGCTCCTGCCCCTCGGG-3’) fused to Nanopore-specific oligonucleotides were used for 16S rRNA and purine permease amplifications, respectively. PCR amplifications of all replicates of the mock bacterial communities (Cn-spiked or not) and corn leaf samples (diseased and healthy) were performed using HotStar Taq Plus DNA polymerase kit (Qiagen, Canada) and barcoded using the SQK-PBK004 PCR Rapid Barcoding kit (ONT) targeting 16S rRNA and purine permease genes where required. Briefly, a PCR reaction (total volume of 60 μl) consisted of 6 μl QIAGen HotStar 10x Buffer, 12 μl 5x Q-sol, 0.75 μl of 2.5 mM dNTPs, 0.6 μl 20 nM of each primer, 1.8 μl of LWB Barcodes (ONT), 0.6 μl of *Taq polymerase* plus (5U/μl; Qiagen), 6 μl DNA template (5 ng/μl), and 21.65 μl of PCR H_2_O. PCR parameters performed in a TProfesional thermocycler (Biometra, Germany) were an initial denaturation at 95˚C, 5 min; followed by 40 cyles of 95˚C, 30 sec, 65˚C, 30 sec, 72˚C, 60 sec; and a final extension at 72˚C, 5 min. For quality control, 3 μl of the PCR amplicons were verified by agarose (1.3%) gel electrophoresis. The remaining 57 μl of PCR products were purified by Amicon Ultra-0.5 Centrifugal Filters (Millipore, Canada). The DNA concentration of the purified PCR products were quantified by Qubit High Sensitivity DNA assay (ThermoFisher, Canada) as recommended by the manufacturer.

Sequencing reactions were performed on pooled samples using MinION flowcells, FLO-MIN106D (R9.4.1; ONT) connected to a MinION device (MIN20562; ONT). The device was operated by the MinKNOW software (version 1.5.2 and version 1.6.11). Prior to application of the samples, the flowcells were primed with 800 μl of priming solution (30 μl Flushing Tether Solution mixed with 1000 μl of Flushing Buffer; ONT). The pooled amplicon libraries were mixed with 2 μl of PCR H_2_O, 1 μl of Rapid 1D sequencing adapters (RAP; ONT), mixed gently and spun down, and incubated for 5 min at room temperature. Eleven microliters of the barcoded amplicons were added to the loading mix consisting of 34 μl of sequencing buffer (SQB), 25.5 μl of loading beads (LB KIT 1D) and 4.5 μl of PCR H_2_O to a total final volume of 75 μl. The mix was then loaded on the Flowcell via the SpotON port. The manufacturer’s recommended Flowcell protocol was executed through the MinKNOW and run for 12, 6, or 3 hrs depending on the treatment.

### Nanopore sequencing analysis

**Fig 1:**
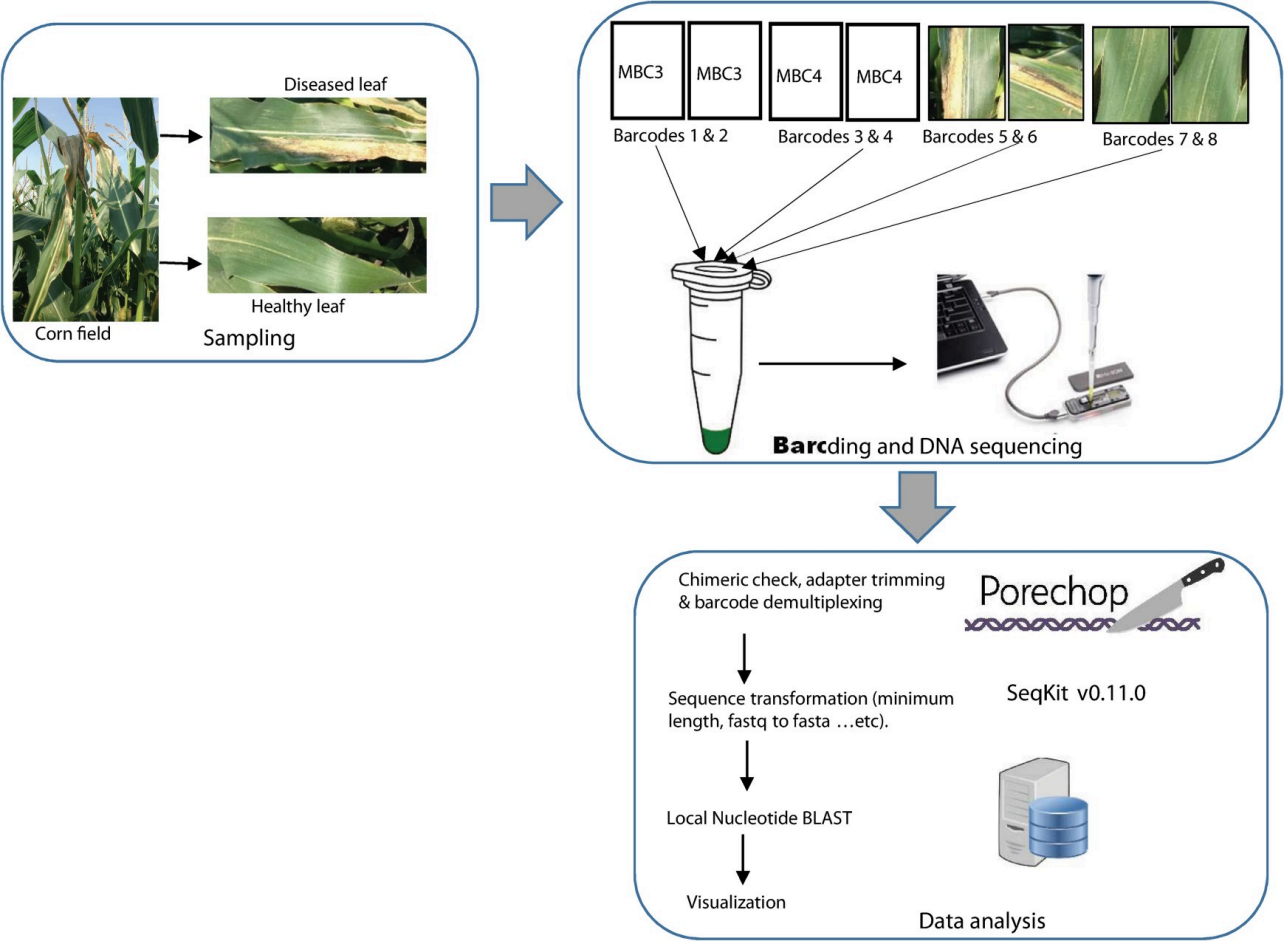
*Clavibacter nebraskensis* Nanopore-based sequencing and detection workflow in mock bacterial communities (MBC) and corn leaf samples from diseased fields, with typical Goss’s wilt symptoms, targeting the purine permease gene. Barcodes **1&2** (MBC3): mock bacterial community spiked with DNA of *C*. *nebraskensis*; barcodes **3 &4** (MBC4): mock bacterial community without spiked DNA of *C*. *nebraskensis*; barcodes **5&6**: diseased leaf samples; and barcodes **7&8**: healthy leaf samples. Detailed processing protocol is given in ‘Material and Methods’ section. High quality reads were taxonomically classified using BLASTn [40] on a custom genome reference database. Image of MinION was downloaded from https://www.mongodb.com/blog/post/oxford-nanopore-technologies-powers-real-time-genetic-analysis-using-docker-mongodb-and-aws.

The raw reads (fast5 files) generated for all the five Nanopore runs were processed using Albacore 2.0.2 software (Oxford Nanopore Technologies) for basecalling. All the fastq files of a Nanopore run were merged into a single fastq file. Barcode de-multiplexing, adapter trimming and quality filtering (QC score > 8) of the merged fastq were done using Porechop (https://github.com/rrwick/Porechop) with the option to discard reads with internal adapters. Seqkit [35] was used to convert the de-multiplexed fastq file into fasta format and to discard reads <1000 nt (1,000–1,500 nt) for 16S rRNA and <300 nt for purine permease fragments. The 16S rRNA derived Nanopore reads were taxonomically analyzed using the Metagenome@KIN software (World Fusion, Tokyo, Japan) with the following parameters: percent identity > 80% and e-value > 1.0 x 10^−30^. Only taxa identified in both replicates of each treatment were used for downstream analyses. Also, Nanopore reads targeting the purine permease gene fragment for specific detection of Cn in serial dilutions, diseased and healthy corn leaf samples, or mock bacterial communities (with or without Cn) were processed. The taxonomic classification was done using a customized local database of all NCBI publicly available genomes of *Clavibacter* and representatives of known bacterial pathogens and saprophytes with a cut-off percent identity of 85%. Nanopore raw sequence raw data are deposited at NCBI Sequence Read Archive (SRA) with accession numbers SRR13149677-SRR13149678 and SRR13150203-SRR13150212. Fig 1 summarizes the detection protocol targeting the purine permease gene.

### MiSeq library preparation, sequencing and analysis

To assess *Pseudomonas* community compositions in the diseased and healthy corn leaf samples, primers were designed and PCR performed targeting a 430-nt variable region of *rpo*D gene, the RNA polymerase sigma 70 factor. The *rpo*D gene has been reported as a reliable species-level taxonomic marker for the genus *Pseudomonas* in previous studies [36, 37].

Library preparation and MiSeq sequencing of *rpo*D gene fragment were performed by the Molecular Technologies Laboratory, Ottawa Research and Development Center (MTL-ORDC, Ottawa, ON, Canada). Briefly, fusion primer cocktails targeting the *rpo*D gene were designed with 0–3 “N” bases in between the Illumina overhang adapter sequence and the gene-specific sequence. This staggering is used to increase base diversity. The pooled total DNA of diseased and healthy leaf samples were PCR-amplified using the fusion primer cocktails, purified with AMPure beads (Beckman Coulter Life Sciences, USA) and normalized using SequalPrep plates (Life Technologies, Canada). A second round PCR was performed to add the Nextera XT indexes (Illumina, Canada), sequencing primer and barcodes. Library sizes were estimated using the Agilent TapeStation D1000 (Agilent Technologies, Germany) assay. Concentrations were calculated using Qubit High Sensitivity DNA assay and KAPA Library Quantification qPCR as recommended by the manufacturer. An equimolar pool was made and loaded on the Illumina MiSeq instrument with 25% PhiX Control Library. The barcoded PCR products were pooled and sequenced with Illumina (Illumina Inc., San Diego, CA, USA) Miseq Nano (500v2, 500 Mb sequencing capacity) at MTL-ORDC (Ottawa, ON, Canada). Illumina MiSeq sequencing raw data are deposited at NCBI Sequence Read Archive (SRA) with accession numbers SRR11799801 and SRR11799802.

FastQC [38] was used to assess the quality of the demultiplexed paired-end (forward and reverse) sequence reads. Poor quality (phred < 20) sequence reads were discarded. The merge script (join_paired_ends.py) in QIIME 1 [39] was implemented to join the paired reads. The obtained reads were screened for a minimum cut-off sequence length of 400 nt using Seqkit software. The quality-filtered reads (> = 400nt) were searched to a custom r*po*D database of over 200 type strains of *Pseudomonas* species based on the following filters: > = 95% match to a database entry and a minimum e-value of 1e^-10^ using the exclude_seqs_by_blast.py command in QIIME. This process binned the sequence reads into matching (known species) and non-matching (potential new genotypes) folders. Matching reads of 178439 and 117398 were obtained for diseased and healthy corn leaf samples respectively. The filtered sequence reads were BLASTn [40] searched to the above mentioned custom database and classified to the corresponding *Pseudomonas* species at 95% similarity and minimum e-value of 1e^-10^. The non-matching sequence reads were assigned to the closest valid *Pseudomonas* species using a lower similarity cut-off threshold of 85% and are referred to as operational taxonomic units (OTUs).

### EvaGreen real-time PCR quantification

Quantitative real-time PCR was used to validate/verify the discrepancies in the relative abundance of *Pseudomonas* community in diseased and healthy leaf samples. EvaGreen^TM^ chemistry was used to quantitate the *Pseudomonas* and *Sphingomonas* communities in total DNA isolated from the leaf samples as previously described [41]. A *Pseudomonas*-specific primer set, F311Ps/R1459Ps (5’-CTGGTCTGAGAGGATGATCAGT-3’ / 5’-AATCACTCCGTGGTAACCGT-3’) targeting the 16S rRNA gene [29] and a *Sphingomonas*-specific primer set, Sphingo-180F/Spingo-420R (5’-GCGTAACGCGTGGGAATCTG-3’/5’-TTACAACCCTAAGGCCTTC-3’), were used. The specificities of the primer sets were confirmed by conventional PCR amplifications, using 7 bacterial genera consisting of 27 strains including *Pseudomonas* and *Sphingomnas* strains. Real-time PCR was conducted in 10-μl reaction volume containing 5 μl of Sso Fast EvaGreen Supermix (Bio-RAD), 0.1 μl of each primer (20 μM) and 1 μl of DNA solution or cell dilution in 1x phosphate buffer solution (PBS; pH = 6.5). Real-time PCR was performed in Chromo4 thermal cycling system (MJ Research, Bio-Rad Laboratories) with optimized amplification conditions for *Pseudomonas* or *Sphingomonas*, respectively, consisting of an initial denaturation at 95 ˚C, 5 or 3 min followed by 40 cycles of 95 ˚C, 30 or 5 sec, 68 ˚C or 63˚C, 30 sec or 10 sec and 72 ˚C or 74 ˚C, 60 ˚C or 15 sec. Standard curves were generated for accurate quantification as previously described [30, 41] using serial cell dilutions of *Pseudomonas canadensis* 2-92^T^ and *Sphingomonas sp*. 23L3C as the reference strains. Triplicates of standards and samples were subjected simultaneously to real-time PCR amplifications with a negative control and blank. Analysis of variance (ANOVA) was performed using the *aov* command in R-statistics (60).

### Estimation of diversity and phylogenetic analysis

Bacterial diversity was assessed using the Shannon’s (*H*) and Simpson’s (*D*) diversity indices, respectively, as implemented using the ‘vegan’ package [42] in R-statistics [43] and Biodiversity calculator (https://www.alyoung.com/labs/biodiversity_calculator.html). Also, the bacterial or *Pseudomonas* communities recovered from diseased and healthy leaf samples were statistically compared using analysis of similarity (ANOSIM) [44] as implemented in mothur [45]. ANOSIM will generate a test statistic, R, used to assess the congruence among individuals grouped according to their respective populations. For phylogenetic analyses, the DNA sequences were aligned using MUSCLE [46] and Neighbor-Joining method [47] was used to infer the trees in MEGA7 [48] with 1000 bootstrap replicates [49]. The evolutionary distances were computed using Jukes-Cantor method [50] in the units of number of base substitutions per site.

## Results

### Specificity of the purine permease DNA fragment and oligonucleotide primers

The comparative analysis of the *Clavibacter* genome nucleotide sequences identified purine permease to be unique to *C*. *nebraskensis* and *C*. *michiganensis* subsp. *californiensis*. None of the other *Clavibacter* species or subspecies have this gene. The presence of this DNA fragment in only Cn and Cmc was confirmed by an extensive BLASTn analysis of publicly available entries of all the four key NCBI databases (nt, refseq_genomes, wgs and ref_prok_rep_genomes). BLAST results based on the nt database retrieved four Cn genomes (strains: NCPPB 2581^T^, 61–1, 7580 and HF4) at a 100% similarity followed by 4 strains of *Curtobacterium* species with percent sequence homologies of 86.98–88.28. Also, *Microbacterium pygmaeum* strain DSM 23142T exhibited a percent sequence homology of 80.12%. With the refseq_genomes and wgs databases, the BLAST hits comprised 3 Cn genomes (strains 2581T or CFBP 7577, DOAB 395 and DOAB 397) at 100% homologies, two genomes of *C*. *michiganensis* strains CFBP 8017 (98.01%) and AY1B2 (97.53%) and Cmc strain CFBP 8216 (97.94%). Alignment of the purine permease nucleotide sequences of all the strains revealed a 19 or 177 single nucleotide polymorphisms (SNPs) between Cn 2581^T^ and Cmc CFBP 8216T or *M*. *pygmaeum* DSM 23142^T^ respectively (S1 Fig). The primer set Cn_pPer11F/ Cn_pPer986R was designed to encapsulate these SNPs for specific detection of Cn especially with a cytosine to adenine polymorphism at position 4 from the 3’-end of primer Cn_pPer11F (S1 Fig). The specificity of this primer set was confirmed using Primer-BLAST with the expected *in silico* amplicon size of 973 bp on all Cn genomes. The reliability of the primer set was validated using conventional PCR amplifications, which showed 973-bp amplicons only with Cn. No PCR products were amplified with 24 other bacterial DNA including all other *Clavibacter* species as well as 14 known bacterial pathogens of corn and saprophytes.

### Nanopore sensitivity and Cn-detection at different run times

**Fig 2:**
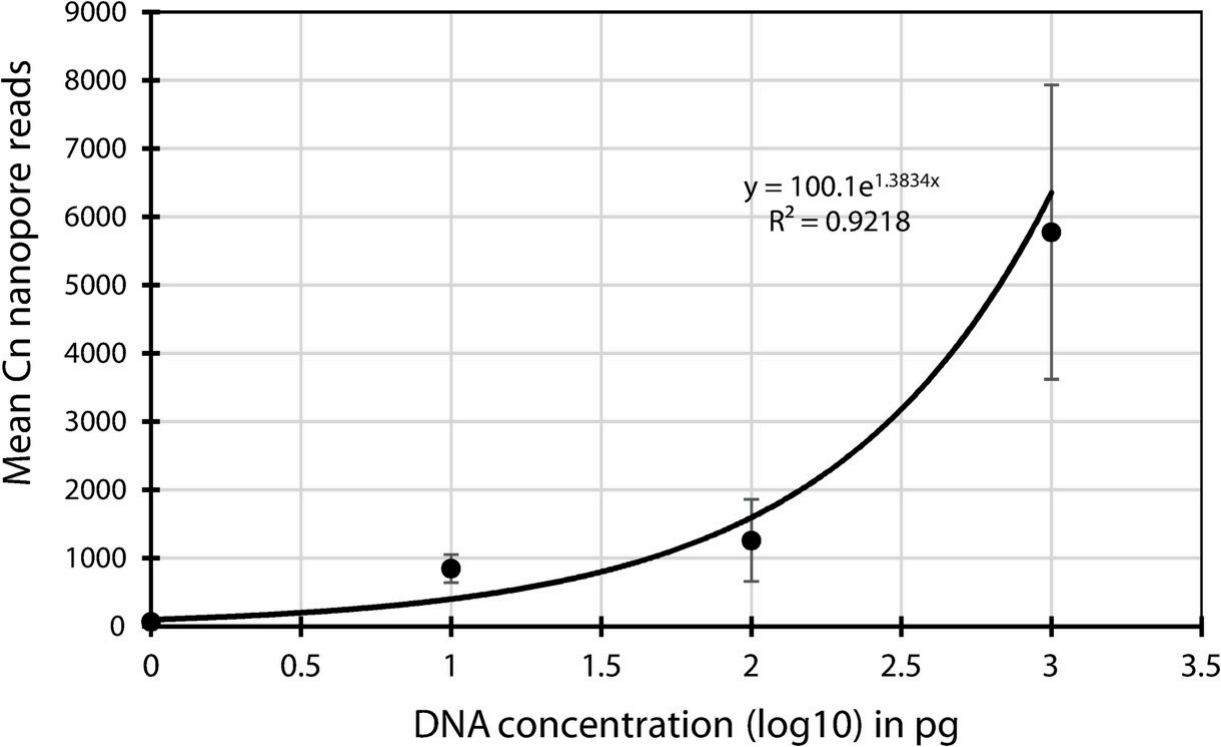
Mean number of MinION Nanopore reads of *Clavibacter nebraskensis* (Cn) DNA spiked, in duplicates, in mock bacterial community of 28 known pathogens and saprophytes of corn after a 3h-run-time MinION Nanopore sequencing. Majority (99.8%) of the Nanopore reads, targeting the purine permease genes, were accurately identified as Cn by a BLAST [2].

The detection limit of the Nanopore-based system was performed on serial dilutions (1, 10, 100 and 1,000 pg/μl) of genomic DNA of *C*. *nebraskensis* NCPPB 2581^T^ spiked in a mock bacterial community of 28 bacterial strains including all *Clavibacter* species and known bacterial pathogens and saprophytes of corn. Fig 2 shows the mean Nanopore reads after a 3-hr run for the four DNA concentrations evaluated. The mean Nanopore reads increased exponentially from 125 reads (1 pg) to about 6,000 reads (1,000 pg) after a 3-hr run time. The exponential increment is represented by the equation y = 100.1e^1.3834x^ with a correlation coefficient of p = 0.960 (r^2^ = 0.923; Fig 2). Over 99.0% of the reads of each DNA concentration were accurately assigned to *C*. *nebraskensis*. A few reads, averaging 1 (1–100 pg/ μl) to 15 (1,000 pg/μl) were attributed to other bacteria including *Clavibacter*. No Nanopore reads were obtained in MBC containing all the other bacterial DNA extracts without the presence of Cn.

**Fig 3:**
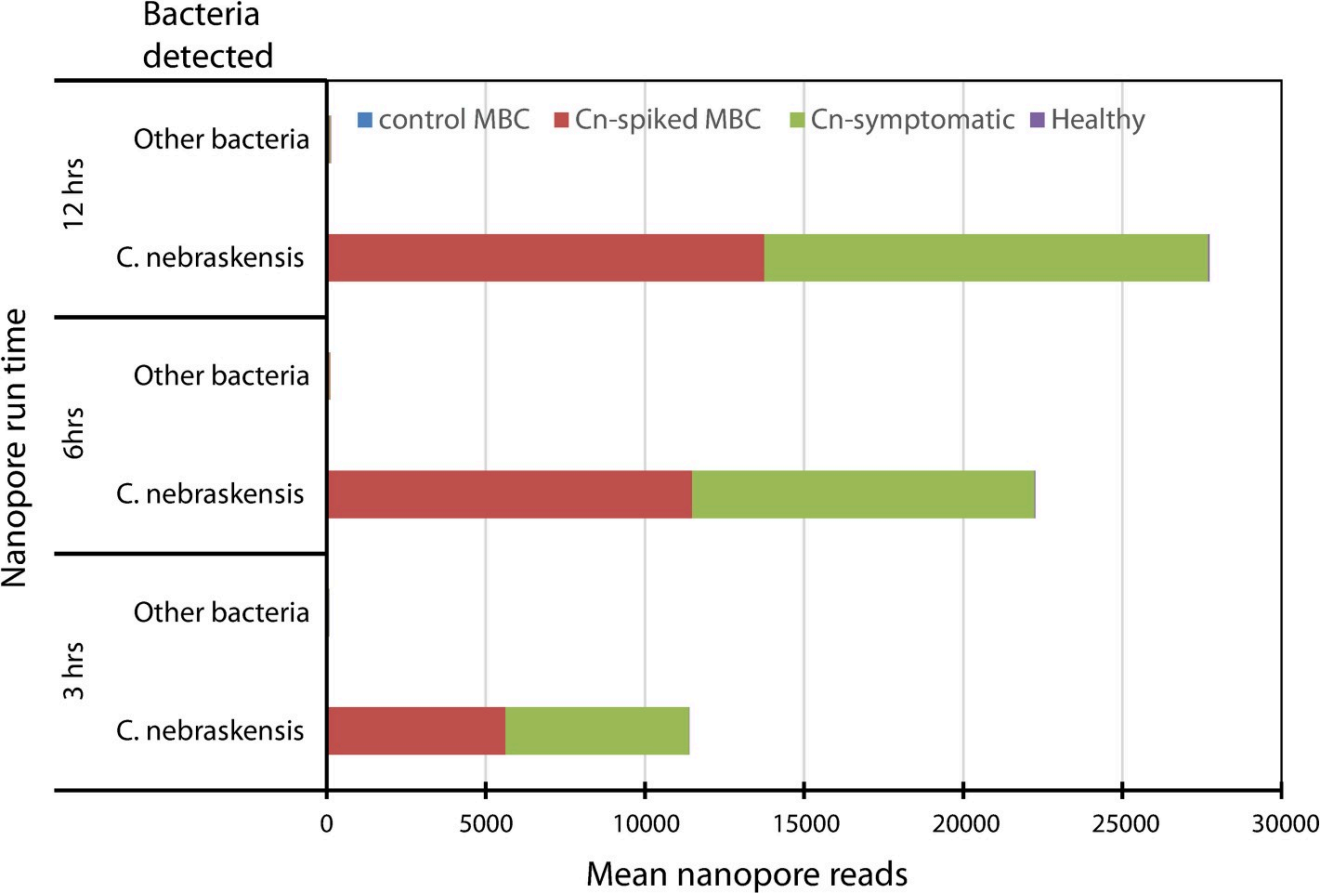
Nanopore-based detection of *Clavibacter nebraskensis* in spiked mock bacterial communities (MBC), naturally infected and healthy leaf samples after 3, 6 or 12 hrs of run-time targeting the purine permease gene fragment. The control MBC (not spiked with *C*. *nebraskensis*) or healthy leaf samples had very low number of Nanopore reads.

Three Nanopore run times were used to process control MBC, Cn-spiked MBC, diseased and healthy leaf samples (Fig 3). The mean Nanopore reads recorded doubled as the run-time increased from 3 to 6 hrs for all the treatments (Fig 3). Increasing the run time from 6 to 12 hrs increased the read counts by only about 20% in all the treatments (Fig 3). The control MBC and healthy corn leaf samples exhibited the lowest number of Nanopore reads, averaging 47 and 14 reads respectively (Fig 3). Cn-spiked MBC and diseased corn leaf samples had high Nanopore read counts with means, across three run times, of 5100, 5000 and 11000 respectively (Fig 3). Over 99.8% of the mean sequence reads recorded for the Cn-spiked MBC and diseased corn leaves were taxonomically assigned to *C*. *nebraskensis* and the rest (0.2%) belonged to other bacteria. Also, 12, 26 and 38 Nanopore reads from healthy corn leaf samples could be taxonomically affiliated to *C*. *nebraskensis*.

### Nanopore-based bacterial profiles in diseased and healthy corn leaves

The 16S rRNA sequences were quality checked and length (1000 bp) filtered to obtain a total of 101,879 and 125,683 reads for diseased and healthy samples respectively. A total of 64,712 (80%) and 108,124 (86.7%) of these reads were classified to the family and genus levels. About 20% and 13.9% of the total number of reads could not be taxonomically classified for diseased and healthy corn leaf samples respectively due to poor quality and/or the presence of chimeras.

**Fig 4:**
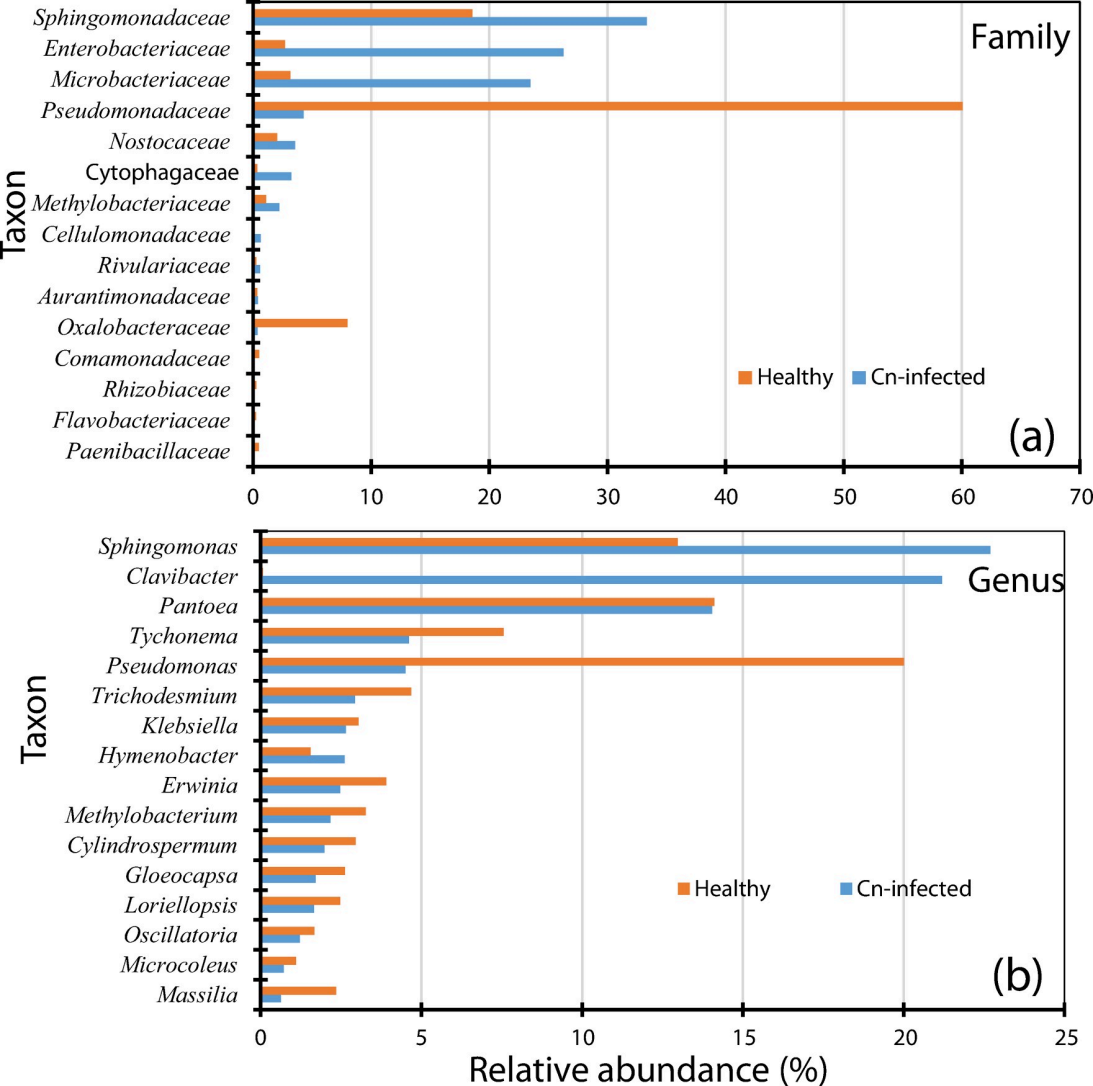
Relative abundance of dominating (>500 Nanopore reads) bacterial families (a) and genera (b) in naturally infected by *Clavibacter nebraskensis* or healthy leaf samples. Note the presence of the genus *Clavibacter* mainly in Cn-infected leaves while the genus *Pseudomonas* was highly abundant in healthy corn leaf samples.

Nanopore reads from healthy samples were taxonomically classified into 14 bacterial phyla, 22 classes, 64 orders, 137 families and 526 genera. Similar taxonomic counts were recorded for diseased samples. The Proteobacteria phylum was the most abundant in both the healthy (80.8%) and diseased (55.0%) leaf samples. The class of Alphaprotebacteria dominated in the diseased leaf samples (30%) while the Gamma-proteobacteria were most prominent in healthy samples (55.1%). Fig 4A shows the relative abundance of the most prominent families in the diseased and healthy leaf samples. *Sphingomonadaceae* (33.7%), *Enterobacteriaceae* (27.0%) and *Microbacteriaceae* (23.0%) were the most prominent families in the necrotic tissues (Fig 4A). *Pseudomonadaceae* (60%), *Sphingomonadaceae* (18.7%) and *Oxalobacteraceae* (8.0%) were the dominant families in healthy leaves (Fig 4A). At the genus-level, *Sphingomonas* (22.7%), *Clavibacter* (21.2%) and *Pantoea* (14.1%) were the most prominent genera while reads taxonomically assigned to the genus *Pseudomonas* made-up only 4.5% (Fig 4B) in diseased leaves. The genus *Pseudomonas* (20.0%) was dominant in healthy leaf tissues followed by *Sphingomonas* (13.0%) while the genus *Clavibacter* had only 0.08% relative abundance (Fig 4B).

Bacterial diversity estimates in healthy and diseased leaf samples based on 16S rRNA showed a total of 377 and 216 genera (richness *S*), respectively (S1 Table). The Shannon’s diversity index was slightly higher in healthy leaf samples (*H* = 3.08) compared to diseased leaves (*H* = 2.80). A similar trend was observed with Simpson’s indices (*D*) of 0.91 and 0.87 for healthy and diseased leaf samples, respectively. Species evenness as expressed by Pielous’s index was identical at 0.52, suggesting that evenness is far from complete in both the healthy and diseased leaf samples. Also, the bacterial community structures were not statistically different.

### Real-time quantification of *Pseudomona*s and *Sphinogomonas* in leaf samples

**Fig 5:**
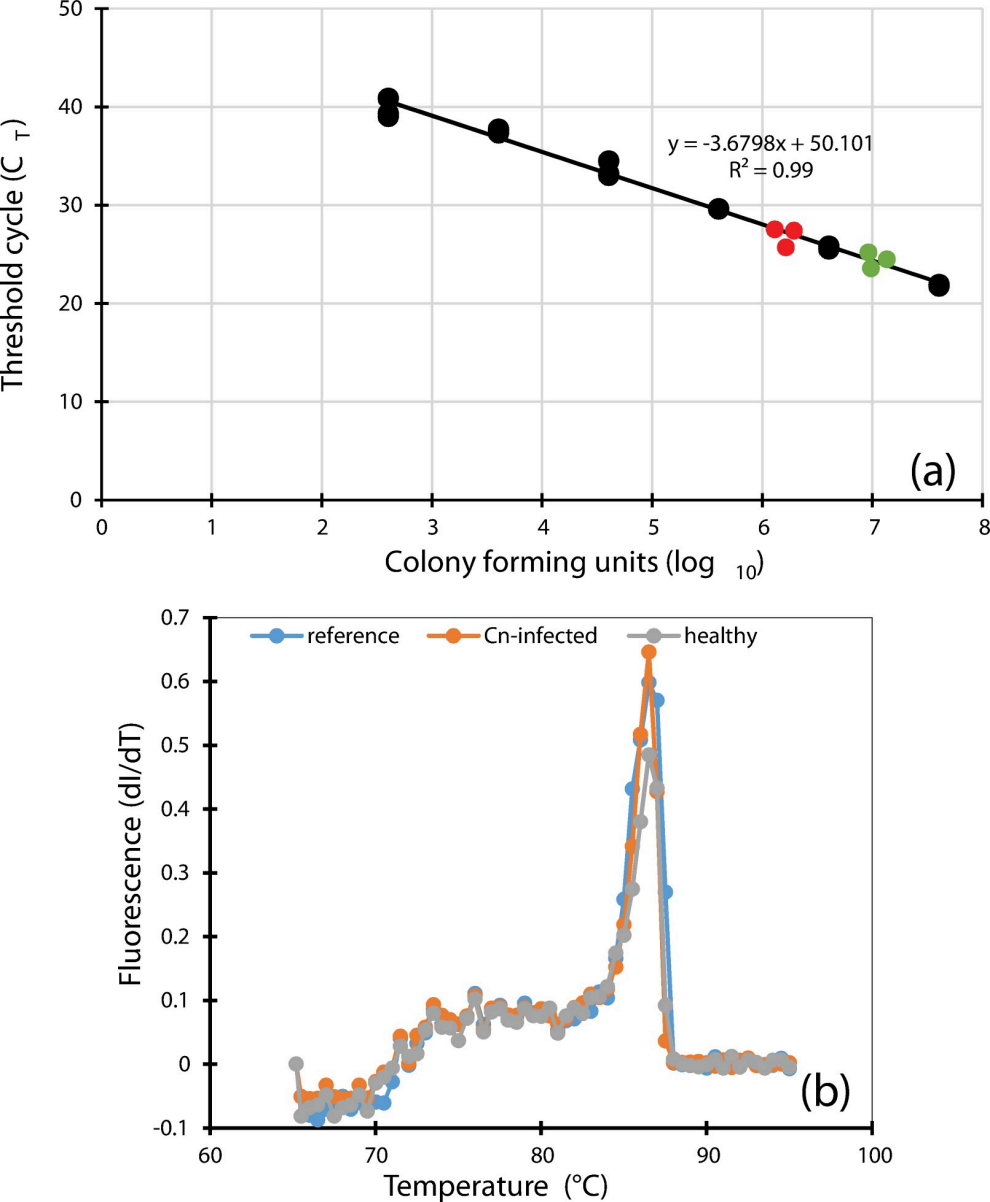
Quantification of *Pseudomonas* populations (a) and melt curve (b) in *Clavibacter nebraskensis-*field-infected corn leaves (green) and healthy leaf samples (red) using EvaGreen qPCR chemistry. *Pseudomonas canadensis* 2-92^T^ was used as reference to generate the standard curve as previously reported [59]. Note that the number of colony forming units present in a sample is inversely proportionate to threshold cycle.

EvaGreen real-time PCR was performed on serial cell dilutions of *Pseudomonas canadensis* 2-92^T^ to generate an optimized standard curve (Fig 5). The threshold cycles (C_T_) increased with each cell dilution (Fig 5A) as the target cell concentration decreased, demonstrating the validity of the assay and showing that quantification of target DNA is possible. An increment in C_T_ value of about 3–4 cycles was recorded as the cell concentration was decreased by 10-fold for each increment (Fig 5A). A linear negative regression, with a coefficient of determination of r^2^ = 0.99, was computed between the threshold cycle (C_T_) and colony forming units (Fig 5A). Healthy leaf samples exhibited a statistically lower mean C_T_ value = 24.00±0.25 (p = 0.0015**) while diseased samples had a mean C_T_ value of 26.57±0.53. Fig 5A shows the position on the standard curve of the recorded C_T_ values for healthy (green circles) and diseased (red circles) leaf samples. This corresponds to a significant (p = 0.001**) higher number of cells, 1.14 x 10^7^ CFU/ml, in healthy leaf samples, indicating a 4.54 times higher number of *Pseudomonas* cells compared to diseased leaf samples (2.51 x 10^6^ CFU/ml). Only one amplicon of the expected size was recorded using agarose gel electrophoresis, confirming the specificity of the primer set. A melt curve analysis confirmed the dissociation characteristics of the double-stranded amplicon obtained at the expected melting temperature of about 86.5°C (Fig 5B).

For EvaGreen quantification of the cells of *Sphingomonas* in the samples, *Sphingomonas sp*. strain 23L3C was used as reference. A standard curve of serial cell dilutions gave a linear negative correlation between the C_T_ values and the log number of *Sphingomonas* cells with a coefficient of determination (r^2^) of 0.99 (S2A Fig). Healthy leaf samples exhibited a statistically (p = 0.013*) higher mean C_T_ value = 25.51±0.35 (green circles) while diseased samples had a mean C_T_ value of 23.94±0.12 (red circles), suggesting a statistically (p = 0.05) higher number of *Sphingomonas* cells in the latter. This corresponds to 1.27 x 10^6^ CFU/ml (p = 0.011*) in healthy leaf samples compared to diseased samples (2.87 x 10^6^ CFU/ml; S2A Fig). A melt curve analysis confirmed the dissociation characteristics of double-stranded amplicon obtained at the expected melting temperature of about 86.5°C (S2B Fig).

### *Pseudomonas* species profile based on *rpo*D Miseq Illumina reads

A total of 410,966 and 294,986 pair-end reads were obtained from the diseased and healthy leaf samples, respectively. About 95% of the raw MiSeq reads were successfully joined by the two ends for each of the samples. After quality check and fragment length (400–440 bp) filtering, 91.3% or 86% of the fastjoined reads were taxonomically assigned to validly described *Pseudomonas* species. A total of 1,615 or 3245 reads from diseased or healthy leaf samples respectively, represented potential novel genotypes. *Pseudomonas* species richness (*S*) in healthy and diseased leaf samples based on *rpo*D gene fragment was 30 or 22 for healthy or diseased leaf samples, respectively. Shannon’s (*H* = 2.32 or 1.890) and Simpson’s (*D* = 0.88 or 0.80) diversity indices were similar in both samples.

**Fig 6:**
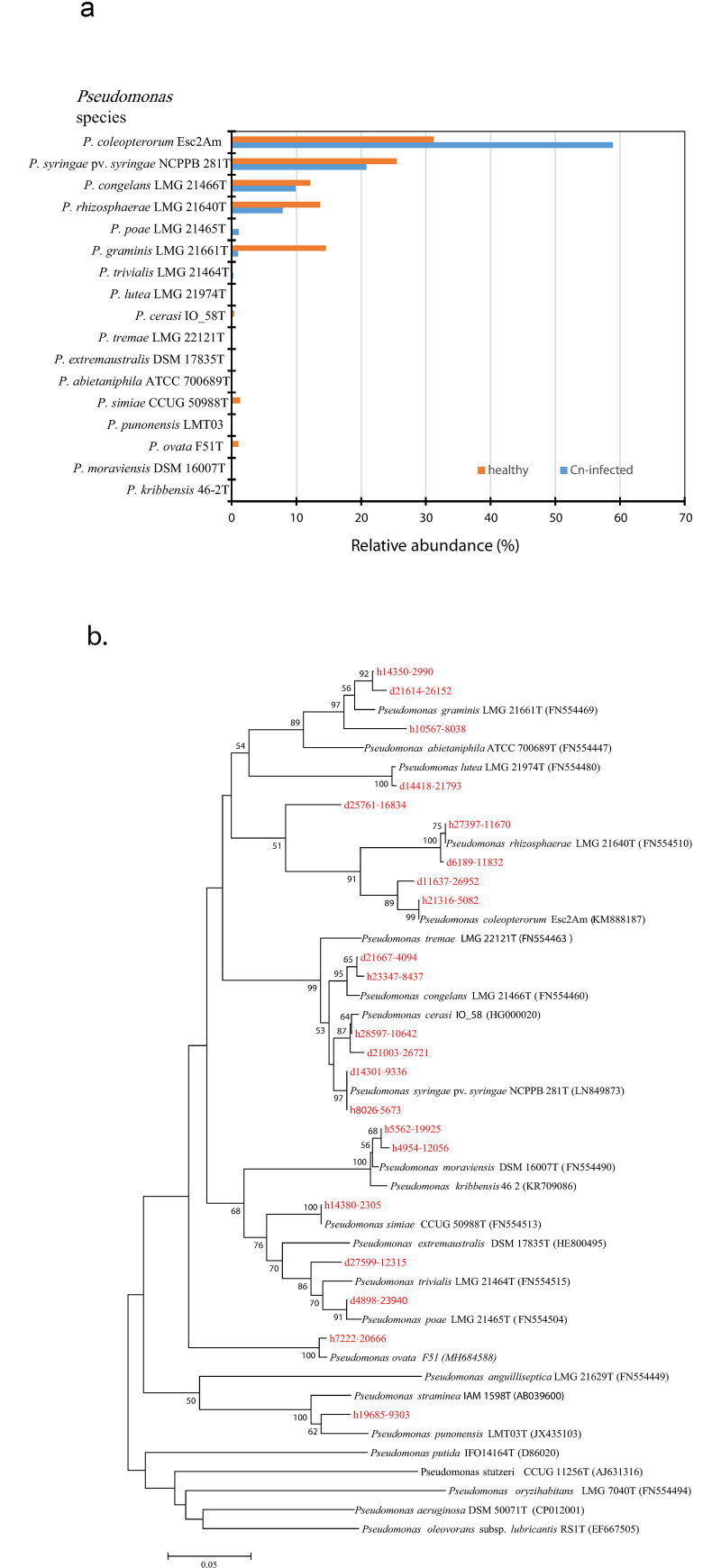
(a) Taxonomic assignment of 400-nt *rpo*D gene sequences derived from diseased and healthy corn leaf samples to validly published *Pseudomonas* species based on BLASTn analysis. *rpo*D gene sequences were obtained using MiSeq technology (Illumina, Inc, Canada); and (b) Neighbor-joining phylogenetic tree showing *rpo*D reads from healthy (h) and diseased (d) samples, in red clustering with validly published type strains of *Pseudomonas* species. The evolutionary distances were computed using the Jukes-Cantor method [30] and are in the units of the number of base substitutions per site. The optimal tree with the sum of branch length = 2.05757992 is shown. Bootstrap (1000 replicates) values >50% are shown next to the branches [16]. Evolutionary analyses were conducted in MEGA7 [33].

Based on a cut-off value of 95% sequence homology, reads derived from diseased and healthy samples could be validly assigned to 12 known *Pseudomonas* species (Fig 6A). *Pseudomonas coleopterorum*, *Pseudomonas*. *syringae* pv. *syringae*, *Pseudomonas congelans*, and *Pseudomonas rhizosphaerae* were dominant (> = 7%) in diseased and healthy leaf samples. *P*. *coleopterorum* showed the highest relative abundance of 58.9% and 31.2% in the diseased and healthy leaf samples respectively; and constitutes the only taxon in which the former had a relative abundance higher than the latter samples (Fig 6A). *Pseudomonas graminis* had a 14-fold higher relative abundance in healthy (14.6%) than in diseased (0.99%) leaf samples (Fig 6A). Each of the samples had 5 *Pseudomonas* species not present in the other samples. Of these unique species, only *Pseudomonas poae* had a relative abundance >1.0% in the diseased leaf samples while *Pseudomonas ovata* and *Pseudomonas simiae* were identified only in healthy leaf samples with relative abundance of 1.05% and 1.3%, respectively (Fig 6A). Fig 6B shows the phylogenetic association of the representative nucleotide sequences of the reads with their taxonomically assigned known *Pseudomonas* species.

**Fig 7:**
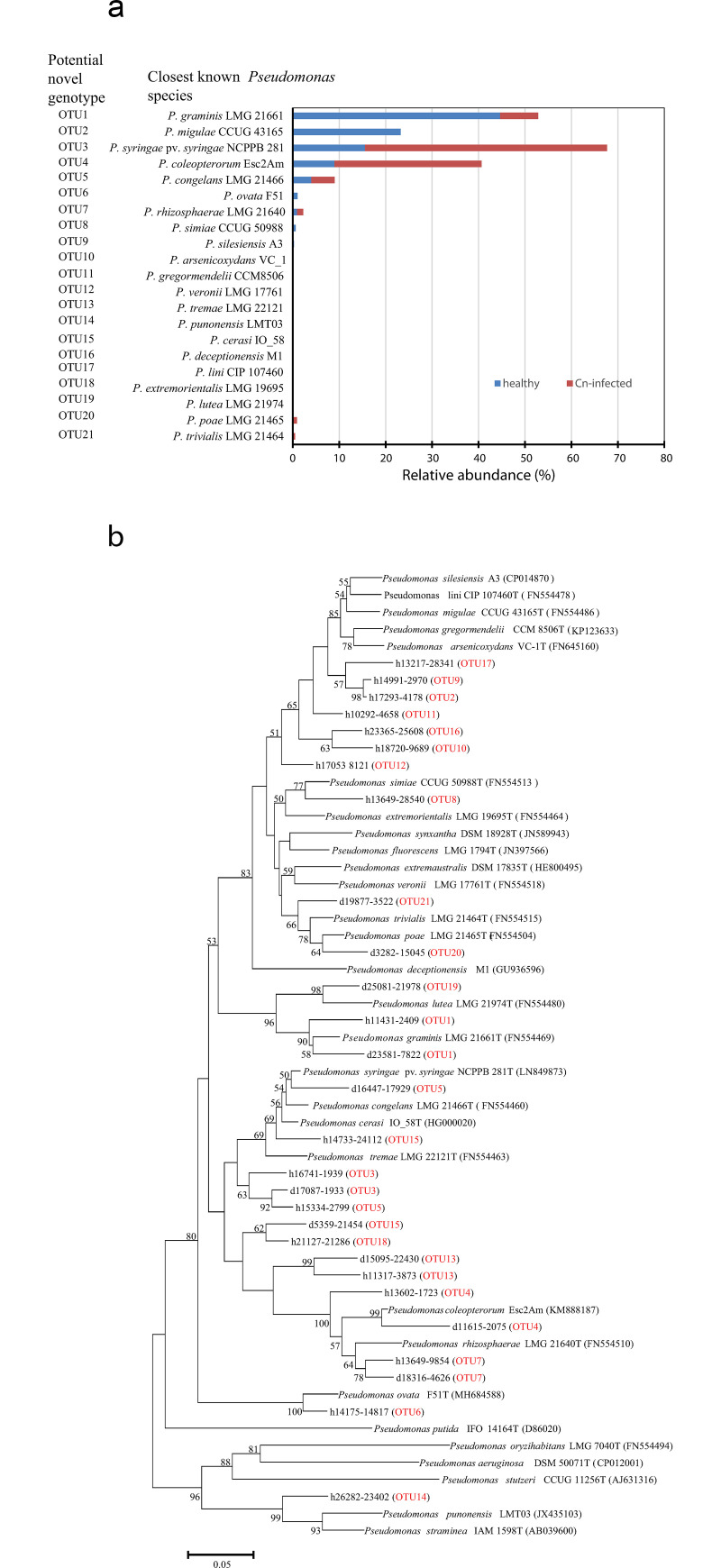
(a) Relative abundance of operational taxonomical units (OTUs) considered as novel genotypes based on nucleotide homology cut-off identity <95% (86.4–94.6%) in healthy and diseased (Cn-infected) leaf samples and the corresponding closest validly described type strain of *Pseudomonas* species; and (b) phylogenetic tree of potential novel *Pseudomonas* genotypes based on a 400-nucleotide *rpo*D gene fragment. OTU codes (red) starting with the letter ‘d’ were from diseased sample while codes with ‘h’ are from healthy leaf samples. Note: OTUs clustering uniquely from validly published type strains. OTUs from the diseased and healthy showing high nucleotide similarities clustered together.

Also, based on homology cut-off of <95%, potential novel genotypes were assigned to 21 OTUs and taxonomically affiliated to the closest type strains of *Pseudomonas* species (Fig 7A). Eighteen of the 21 OTUs were identified in the healthy samples with 11 unique to this sample type (Fig 7A). The diseased leaf samples had 10 OTUs with only 3 unique to this sample type. OTU1, the closest type strain being *P*. *graminis*, was dominant in the healthy leaf samples had 5.5-fold relative abundance over diseased leaf samples (Fig 7A). OTU3 (closest to *P*. *syringae* pv. *syringae*) and OTU4 (*P*. *coleopterorum* as the closest type strain) were more abundant (about 3.5-fold) in diseased leaves than in healthy samples (Fig 7A). The proportions of OTU5, *P*. *congelans* as the closest know species, were similar in both samples. OTU2, the second most abundant OTU in healthy leaf samples (23.3%) and affiliated to the type strain of *P*. *migulae*, was absent in diseased corn leaves (Fig 7A). Phylogenetic analysis of representative nucleotide sequences confirmed the uniqueness of these genotypes as the representative *rpo*D sequences clustered distantly and uniquely within the genus *Pseudomonas* (Fig 7B).

## Discussion

This study developed and validated the first MinION Nanopore-based workflow for accurate and reliable detection of *C*. *nebraskensis*, the Goss’ bacterial wilt and blight pathogen of corn. In addition, bacteriomic profile analysis using MinION Nanopore 16S rRNA reads revealed a significantly low relative abundance of *Pseudomonas* (4.5%) in diseased leaf samples compared to a 20.0% in healthy leaves. We employed EvaGreen real-time PCR to investigate this discrepancy as well as using MiSeq sequencing targeting *rpo*D gene to profile *Pseudomonas* species present in diseased and healthy corn leaf samples.

The Oxford MinION sequencer is a pocket-sized device that allows for real time long read and rapid sequencing of nucleic acids [21, 51, 52]. Its application in the medical field e.g. clinical diagnosis of infectious diseases is increasing [24, 27, 53–55]. Nanopore sequencer has been used to accurately identify bacterial and viral pathogens in clinical samples [24, 56, 57]. The advantages of the MinION Nanopore technologies include comparatively low pricing, its portability, and great potential for real-time analysis of data [2, 21, 55] as well as low laboratory space requirement. Despite its use, the current drawbacks of the technology include a comparatively low per read accuracy of about 90%[22]. Despite a high number of reported applications of the MinION nanopore in human clinical diagnosis, only few reports [2, 25, 28] exist for its use in diagnosing plant diseases. Chalupowicz et al. [28] demonstrated the detection of several phytobacterial pathogens and Hu et al. [2] diagnosed wheat fungal diseases on artificially inoculated plants and nurseries, respectively. Boykin et al. [25] and Faino et al. (unpublished) are the few reported studies that used naturally infected plants for detection/diagnosis of the cassava virus and *Xylella fastidiosis*, respectively, using MinION Nanopore sequencer. No reports have been published on the use of the Nanopore technology for diagnosis of the Goss’s bacterial wilt pathogen (*C*. *nebraskensis*) of corn.

We report an amplicon Nanopore-based detection system for *C*. *nebraskensis*, an A2 quarantine bacterial pathogen based on the categorization of European and Mediterranean Plant Protection Organization. The system, at a 3-hr run time and targeting the purine permease gene, reliably detected Cn DNA in spiked mock bacterial community at 1-pg sensitivity levels which is comparable with real-time PCR assays [5, 30, 58]. The results presented here also show that the number of Nanopore reads increased with increasing sequencing run-time, suggesting that DNA amounts less than 1 pg might be detected by doubling the run-time from 3 hr to 6 hr and as such providing an added advantage over real-time PCR assays. This system also successfully detected the presence of *C*. *nebraskensis* in naturally-infected corn leaf samples collected in 2015 and 2016. Positive detections (>5000 reads) of Cn were achieved at 3-hr, 6-hr or 12-hr Nanopore run-time in all samples that were previously determined to be positive using TaqMan real-time PCR [5]. This corroboration of real-time PCR results is a direct validation of the reliability of the Nanopore-based system. The samples that tested negative using real-time PCR [5] generated a negligible number of reads (<20) which could be a result of error in de-multiplexing or a few cells on the leaf surfaces under field condition. This, also, suggests that the process from DNA extraction to PCR amplification produced high-quality DNA with minimum PCR inhibitors as confirmed using lambda-based DNA TaqMan real-time assay as previously reported [30].

16 rRNA Nanopore sequencing and analysis were also performed to profile the bacterial communities of the diseased compared to healthy leaf tissues. The following bacterial families *Sphingomonadaceae*, *Enterobacteriaceace*, *Microbacteriaceae*, *Pseudomonadaceae* and *Oxalobacteriaceae*, were dominant in diseased and healthy leaf tissues. At the genus-level, however, *Clavibacter* was only profiled in diseased leaf tissues; and based on the Nanopore system developed here and the TaqMan real-time PCR [5], this could be attributed to the presence of *C*. *nebraskensis*. Also, the genera *Pantoea* and *Erwinia*, known to encompass corn pathogens, were more prominent in diseased leaf samples, suggesting potential co-infection/co-existence. The nature of this relationship is still to be elucidated. It is, however, possible that in commensal co-habitation the presence of one pathogen might create favourable micro-conditions for the establishment of another pathogen. For all the genera profiled, the diseased leaf samples had comparable relative abundance to healthy leaf tissues with the exception of the genera *Clavibacter* and *Pseudomonas*. The proportion of reads classified to the genus *Pseudomonas* is about 4.4x in healthy than diseased leaf samples. The only significant change between the bacterial profiles of healthy and diseased samples is the colonization of the leaves by *C*. *nebraskensis* and as such it could be suggested that this caused a decreased in *Pseudomonas* community size. The significantly low relative abundance of *Pseudomonas* in diseased samples was corroborated by qPCR using EvaGreen chemistry. This is an interesting phenomenon that needs further investigation to understand the mechanism(s) involved in this interaction. *C*. *nebraskensis* is not known to produce wide-spectrum antimicrobial compounds that are effective against known *Pseudomonas* species. Like most bacteria, *C*. *nebraskensis* produces bacteriocins that are effective against closely related species such as *C*. *michiganensis* [59]. In the contrary, pseudomonads are known producers of potent antimicrobial compounds. For example, 2,4-diacetylphloroglucinol-producing *Pseudomonas* species inhibit the growth of *Clavibacter michiganensis* subsp. *michiganensis* [60]. It was, thus, worthwhile to look at the *Pseudomonas* community in diseased and healthy leaves for potential explanation(s). There was no difference with regards to the number of identified core known *Pseudomonas* species as indicated by the Shannon’s and Simpson’s diversity indices (data not shown). However, the proportion of *rpo*D MiSeq reads classified as *P*. *graminis* LMG 21661^T^ is 14 times higher in healthy than in diseased leaf samples. This is also true with respect to potential novel genotypes in which the proportion of *rpo*D MiSeq reads associated with OTU1 (closest known species being *P*. *graminis*) in healthy leaves is 44.7% compared to 8.2% in diseased samples. In addition, OTU2 (closest known species, *Pseudomonas migulae*), the second most abundant OTU in healthy leaves was not recorded in diseased leaf samples. We also showed that most of the plant pathogenic *Pseudomonas* species, e.g. *P*. *syringae*, *P*. *poae*, *P*. *tremae*, *P*. *lutea* and *P*. *trivialis*, were dominant in diseased samples while non phytopathogenic species (*P*. *ovata*, *P*. *rhizosphaerae*, *P*. *simiae*, *P*. *kribbensis*, *P*. *moraviensis* and *P*. *punonensis*) were dominant in healthy leaves. We hypothesize a potential *Pseudomonas*-*Pseudomonas* interaction within the necrotic lesions which provide a moist and nutrient-limiting environment. The ability for pathogens to survive in competitive conditions for nutrients, e.g. diseased lesion tissues, is an important ecological fitness factor. The production of antimicrobial compounds is one of the strategies as this affects population dynamics [61–63]. It is possible that the production of bacteriocins (proteinaceous substances synthesized ribosomally, and usually targeting closely-related species) could be one of the possible explanations for the low *Pseudomonas* counts in diseased samples since the micro-niche of the necrotic lesions favors nutrient competition. *Pseudomonas syringae* pv. *syringae* is known for its production of potent bacteriocins against some strains or species groups of *P*. *fluorescens*, *P*. *putida*, *P*. *aeruginosa* and even other *P*. *syringae* [61, 64, 65]. The production of the bacteriocins by *P*. *syringae* pv. *syringae* and/or other plant pathogenic and saprophytic *Pseudomonas* species in the nutrient-limited lesion niche in diseased leaves might have resulted to an overall reduction in population. It is worthwhile noting that the population of *P*. *syringae* pv. *syringae* did not significantly change between the samples, thus, supporting the above hypothesis. Also, *P*. *coleopterorum*, a cellulase-producing bacterium [66], was the dominant species in diseased leaves in about 2:1 ratio compared to healthy leaf samples. Its relatively high proportion in leaves colonized by Cn (diseased samples) is not surprising given that the dead cells that constitute the lesions provide plant residues required by cellulose-degrading microbes such as *P*. *coleopterorum*. Its high activity might be detrimental to other *Pseudomonas* species. Antimicrobial molecules produced by members of a given community play a significant role in microbiome structuring, however, it is still unclear how communities are shaped by specific factors [67, 68].

In conclusion, the developed Nanopore-based detection system provides a rapid way for diagnosis of the disease at the same sensitivity level of 1 pg as reported by real-time PCR [5, 58]. Its relatively low cost, portability and real-time data analysis are attractive advantages over the real-time PCR technology. The potential for automation of the data analysis and decision-making for the presence or absence of pathogens based on cut-off read counts could revolutionize plant disease monitoring and surveillance as well as minimizing human errors. The reliability was evaluated on naturally-infected corn leaves collected in 2015 and 2016 that were confirmed to be positive or negative using TaqMan real-time PCR [5]. This suggests that the system is robust and could be used for forensic investigations dealing with aged residues. Also, Nanopore-based bacteriomic profile analysis revealed statistically similar bacterial communities in diseased and healthy leaf samples. However, significantly low relative abundance of Nanopore reads taxonomically assigned to the genus *Pseudomonas* in diseased compared to healthy leaf samples is reported. This is corroborated by qPCR EvaGreen chemistry. The Nanopore-based system developed here could be a useful tool for diagnosis of the Goss’s bacterial wilt and blight disease (GBWD) by direct processing of corn leaves. Finally, this study provides the first insight on *Pseudomonas* population dynamics in necrotic leaf lesions of GBWD.

## Supporting information

S1 Fig Alignment of partial purine permease nucleotide sequences showing mismatches and primer positions sequences: 1, *Clavibacter nebraskensis* NCPPB 2581; 2, *Clavibacter nebraskensis* 61–1; 3, *Clavibacter nebraskensis* 7580; 4, *Clavibacter nebraskensis* HF4; 5, *Clavibacter nebraskensis* DOAB 395; 6, *Clavibacter nebraskensis* DOAB 397; 7, *Clavibacter michiganensis* CFBP 8017; 8, *Clavibacter michiganensis* subsp. *californiensis* CFBP 8216; 9, *Clavibacter michiganensis* AY1B2; and 10, *Microbacterium pygmaeum* DSM 23142^T^. (PDF)

S2 Fig Quantification of *Sphingomonas* populations (**a**) and melt curve (**b**) in diseased corn leaves (D_dI/dT; red) and healthy leaf samples (H_dI/dT; green) using EvaGreen qPCR chemistry. *Sphingomonas sp*. 23-L3C was used as reference to generate the standard curve as previously reported [41]. Note that the number of colony forming units present in a sample is inversely proportionate to threshold cycle.
(PDF)

S1 Table Bacterial (16S rRNA) and *Pseudomonas* (*rpo*D) species diversity indices. (PDF)
