# Supplementary figures and images for: MinION Nanopore-based detection of Clavibacter nebraskensis, the corn Goss’s wilt pathogen, and bacteriomic profiling of necrotic lesions of naturally-infected leaf samples
Source: PLoS One. 2021 Jan 22;16(1):e0245333. doi: 10.1371/journal.pone.0245333 (PMC7822522; doi:10.1371/journal.pone.0245333)

Xu\_et\_al.(2020) PloS One S1\_Fig

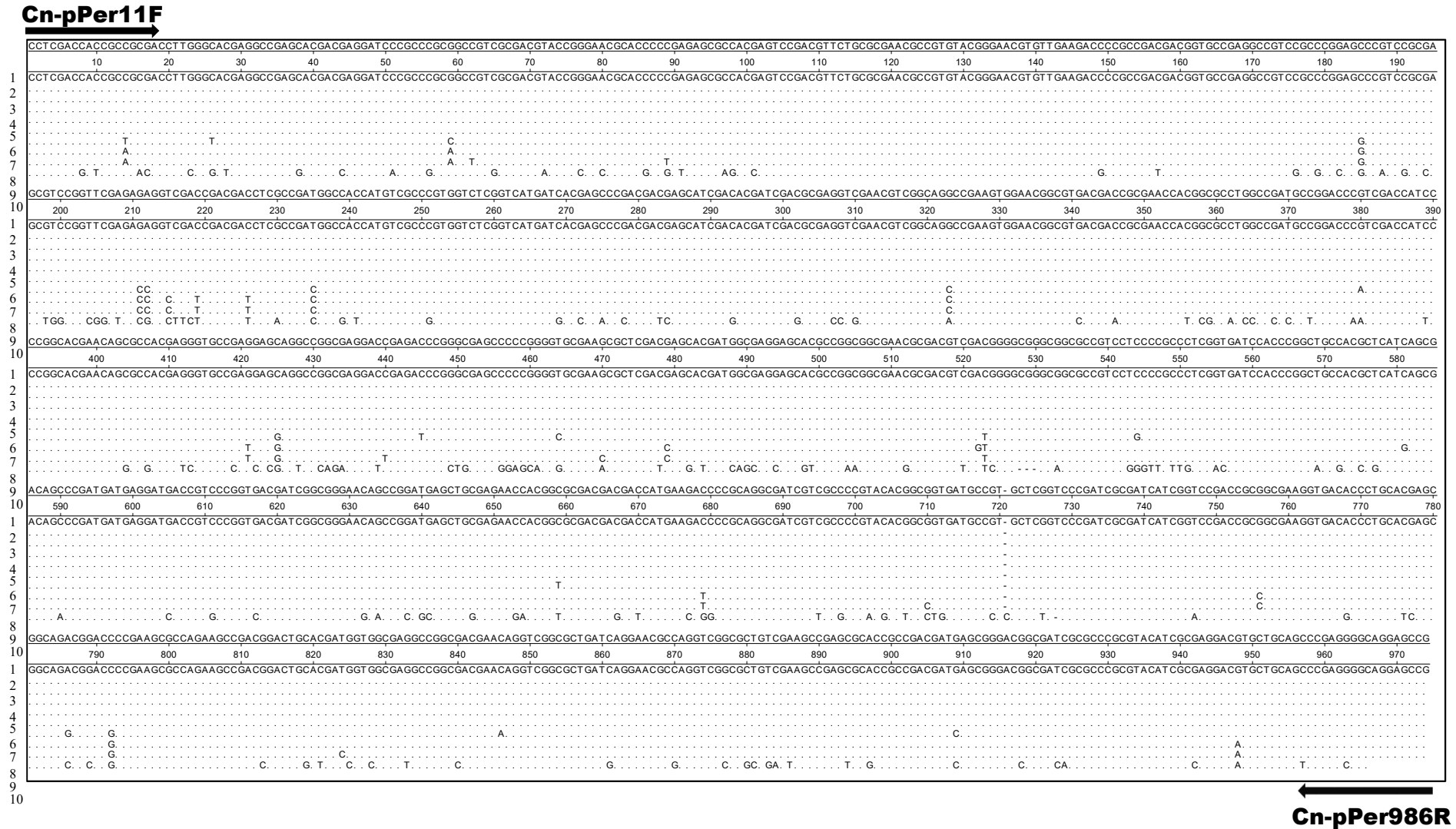

Supplement: S1 Fig — (PDF) [file pone.0245333.s001.pdf]

*Sphingomonas*

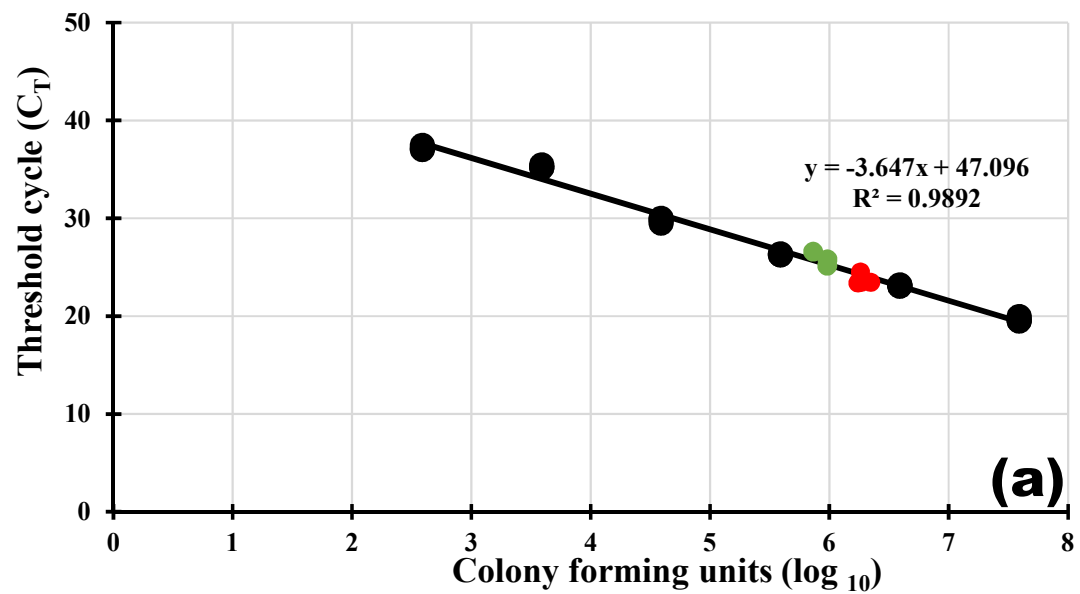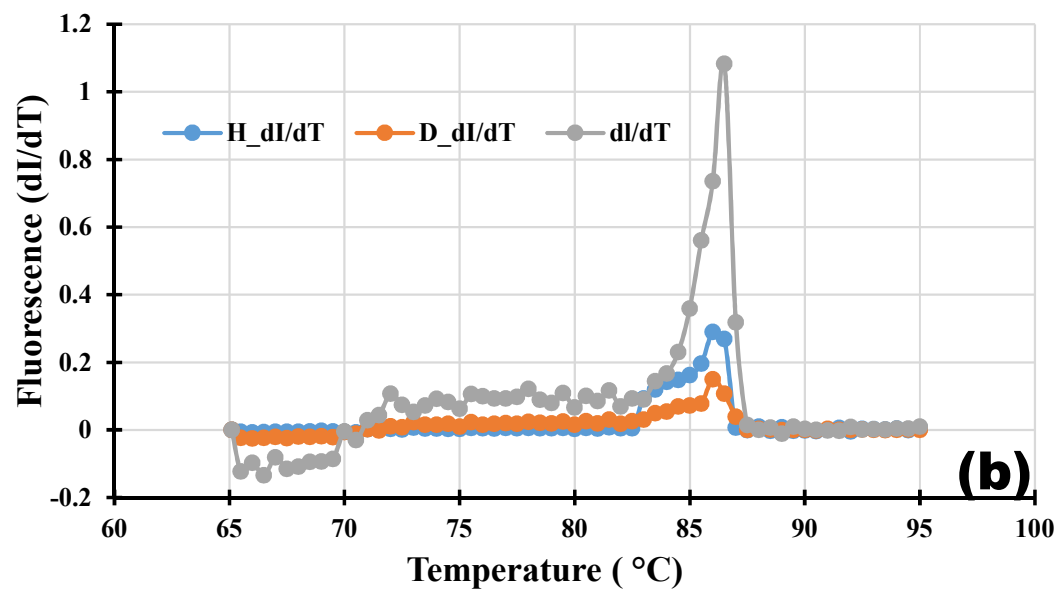

Supplement: S2 Fig — Quantification of Sphingomonas populations (a) and melt curve (b) in diseased corn leaves (D_dI/dT; red) and healthy leaf samples (H_dI/dT; green) using EvaGreen qPCR chemistry. Sphingomonas sp. 23-L3C was used as reference to generate the standard curve as previously reported [41]. Note that the number of colony forming units present in a sample is inversely proportionate to threshold cycle. (PDF) [file pone.0245333.s002.pdf]
