# Supplementary material for: MinION Nanopore-based detection of Clavibacter nebraskensis, the corn Goss’s wilt pathogen, and bacteriomic profiling of necrotic lesions of naturally-infected leaf samples
Source: PLoS One. 2021 Jan 22;16(1):e0245333. doi: 10.1371/journal.pone.0245333 (PMC7822522; doi:10.1371/journal.pone.0245333)
Supplement: S1 Table — (PDF) [file pone.0245333.s003.pdf]

S1 Table. Bacterial (16S rRNA) and *Pseudomonas* (*rpo* D) species diversity indices

	Healthy leaves	Diseased leaves
**Bacterial diversity:**		
Shannon-Weiner's index (*H*)	3.08	2.8
Simpson's index (D)	0.91	0.87
Simpson's reciprocal index (1/1-D)	10.85	7.99
Fisher's alpha index[a]	46.26	29.56
Species richness (*S*)	377	216
Pielou's eveness (*J*)	0.52	0.52
***Pseudomonas* species diversity:**		
Shannon-Weiner's index (*H*)	2.32	1.89
Simpson's index (*D*)	0.803	0.88
Simpson's reciprocal index (1/1-D)	8.18	5.07
Fisher's alpha index*	2.18	1.57
Species richness (*S*)	30	22
Pielou's eveness (*J*)	0.68	0.61

[a]A parametric divcersity index that assumes that the species abundance is modulated as log series distribution.
